# Evaluations of coronary microvascular dysfunction in a patient with thrombotic microangiopathy and cardiac troponin elevation: a case report

**DOI:** 10.1093/ehjcr/ytac318

**Published:** 2022-07-29

**Authors:** Kenichiro Otsuka, Yasushi Kono, Kumiko Hirata

**Affiliations:** Department of Cardiovascular Medicine, Fujiikai Kashibaseiki Hospital, 3300-3 Anamushi, Kashiba, Japan; Department of Cardiovascular Medicine, Fujiikai Kashibaseiki Hospital, 3300-3 Anamushi, Kashiba, Japan; Department of Cardiovascular Medicine, Fujiikai Kashibaseiki Hospital, 3300-3 Anamushi, Kashiba, Japan; Department of Medical Science, Osaka Educational University, Kashiwara, Osaka, Japan

**Keywords:** Thrombotic microangiopathy, Thrombotic thrombocytopenic purpura, Coronary microvascular dysfunction, Cardiac troponin, Myocardial infarction with non-obstructive coronary arteries, Case report

## Abstract

**Background:**

Thrombotic microangiopathy (TMA) syndromes include thrombotic thrombocytopenic purpura (TTP) and haemolytic uremic syndrome, and contribute to myocardial infarction and multiple organ failure. Although coronary microvascular dysfunction (CMD) is the key for understanding the pathophysiology of cardiac involvement in TMA, there is limited knowledge on the recovery from CMD in patients with TMA.

**Case summary:**

An 80-year-old woman was brought to the emergency department due to worsening back pain, dyspnoea on exertion, jaundice, and fever. Although she had typical TTP symptoms and elevated cardiac troponin level, ADAMTS13 activity was preserved (34%), leading to the diagnosis of TMA with myocardial infarction. She underwent plasma exchange and was administered aspirin and prednisolone. Magnetic resonance imaging revealed iliopsoas abscess, which is a possible aetiologic factor of sepsis-related TTP. She had impaired coronary flow reserve (CFR) with angiographically non-obstructive epicardial coronary arteries. Improved CFR was observed on follow-up, suggesting existence of transient CMD caused by TMA. After treatment of the iliopsoas abscess with antibiotics for 3 months, she was discharged without any adverse complications.

**Discussion:**

Coronary microvascular dysfunction is an underlying mechanism of myocardial infarction, with or without epicardial obstructive coronary artery stenosis. TMA is characterized by pathological lesions caused by endothelial cell damage in small terminal arteries and capillaries, with complete or partial occlusion caused by platelet and hyaline thrombi. CMD and its recovery are keys for understanding the natural history of cardiac involvement in TMA. *In vivo* evaluations of CMD can provide mechanistic insights into the cardiac involvement in TMA.

**Learning points**

- Thrombotic microangiopathy (TMA) can be life-threatening, and its underlying mechanisms involve platelet aggregation and increased mechanical stress in the microcirculation.
- Coronary microvascular dysfunction (CMD) caused by TMA contributes to multiple organ failure, myocardial infarction/injury, and sudden cardiac death.
- Non-invasive assessment of coronary microvascular function and its recovery can provide mechanistic insights into the cardiac involvement in TMA.

## Introduction

Thrombotic microangiopathy (TMA) is a spectrum of life-threatening syndromes, including thrombotic thrombocytopenic purpura (TTP) and haemolytic uremic syndrome (HUS).^1^ The mechanisms underlying TMA involve platelet aggregation and increased mechanical stress in the microcirculation that contribute to multiple organ failure, including renal and heart failure, myocardial infarction/injury, and sudden cardiac death.^1,2^

Myocardial infarction/injury is defined by the presence of cardiac troponin elevation caused by structural or functional obstruction of the coronary arteries.^3^ In a single-centre study, Wahla *et al*.^2^ demonstrated 15.3% of the patients diagnosed with TTP (*n* = 85) had myocardial infarction. Furthermore, myocardial infarction of the non-obstructive coronary arteries (MINOCA) can also cause cardiac troponin elevation.^3,4^ Although assessment of the coronary microvascular function is important for understanding the pathophysiology of TMA, there are limited reports on *in vivo* evaluations of coronary microvascular dysfunction (CMD) and its recovery. Herein, we describe a case of cardiac troponin elevation with CMD associated with TMA, demonstrating non-invasive assessment of the recovery of the coronary microvascular function.

## Timeline

**Table:** 

Admission	
Day 1	Treatment with bi-level positive airway pressure (BiPAP), carperitide, diuretics, and antibiotics in the intensive care unit. Blood culture and urine cultures were performed.
Day 2	Transient neurologic deficits. Fresh frozen plasma (FFP) infusion.
Day 3	Treatment with steroid pulse therapy (1 g/day for 3 days), and plasma exchange.
Day 5	BiPAP free, treatment with aspirin.
Day 6	Prednisolone 40 mg/day.
Day 7	Transthoracic Doppler echocardiography (TTDE) revealed impaired coronary flow reserve (CFR = 1.7). No evidence of an acute infarct area on cardiac magnetic resonance (CMR) imaging.
Day 21	Plasma exchange free, prednisolone 30 mg, renal function improved, move out from high-care unit.
Day 22	Invasive coronary angiography.
Day 30	Follow-up TTDE demonstrated improved CFR of 2.5.
Day 35	Prednisolone 20 mg/day (tapered by 5 mg/week).
Day 50	Prednisolone 10 mg/day (tapered by 2.5 mg/2 weeks).
Day 90	Antibiotics free, blood culture negative.
Day 120	Discharged with prednisolone 5 mg/day after rehabilitation.

## Case presentation

**Figure 1:**
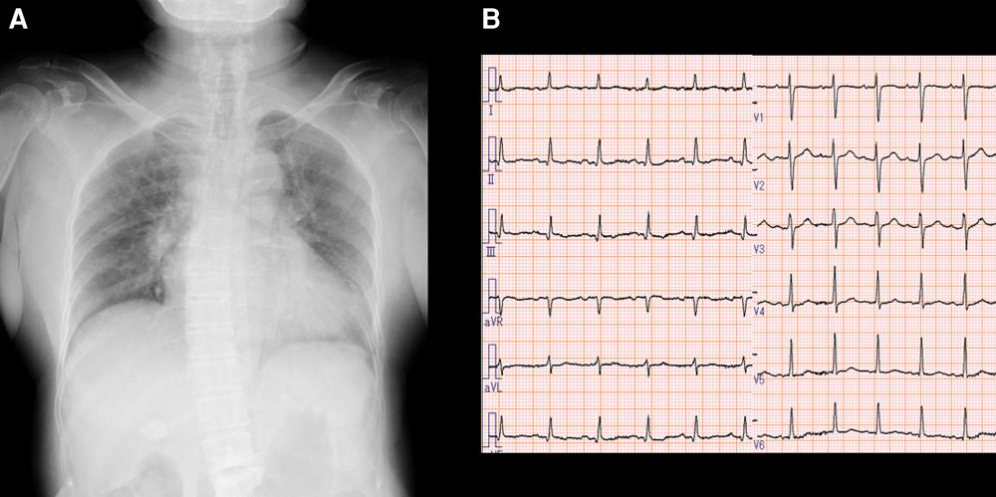
X-ray and electrocardiogram on admission. (*A*) X-ray depicting bilateral congestion. (*B*) Twelve-lead echocardiogram at admission showing ST depression in V5 and V6 leads, with sinus tachycardia and heart rate of 105 b.p.m.

An 80-year-old woman presented to the emergency department with a 2-week history of worsening back pain, a 5-day history of dyspnoea on exertion, and high temperature. Approximately 2 weeks before admission she had an episode of back pain and high temperature, and 2 days before admission, she had transient unconsciousness and elevated temperature (40°C). She was a passive smoker on oral antidiabetic medication, and had no documented medical history of prior TTP or autoimmune disorder. On physical examination, she was conscious, had dyspnoea without chest pain, a body temperature of 38.2°C, blood pressure of 106/72 mmHg, pulse of 110 b.p.m. with 90% oxygen saturation (room air), and bilateral rales; regular heart rhythm; II/VI systolic murmur at apical; distension of the neck veins; jaundice; and petechiae and purpura in her toes. A neurological examination revealed no focal neurologic deficit. Laboratory tests (Table 1) revealed decreased platelet count of 13 000/μL and estimated glomerular filtration rate of 22 mL/min/1.73 mm^2^, increased level of total bilirubin of 7.3 mg/dL, increased level of lactate dehydrogenase (LDH) of 812 IU/L, and elevated levels of brain natriuretic peptide and cardiac enzymes [411 IU/L creatine kinase (CK), 40 IU/L CK-MB, and 19 966 pg/mL cardiac troponin I]. Chest radiography revealed congestion (*Figure 1A*), and a 12-lead electrocardiogram on admission showed ST depression in V5 and V6 leads with normal sinus rhythm and a heart rate of 105 b.p.m. (*Figure 1B*). TTDE revealed a preserved left ventricular ejection fraction (50%) with hypokinesis of the inferior-posterior wall, suggesting non-ST-segment myocardial infarction. Chest computed tomography (CT) examination revealed lung congestion but no signs of infection. Brain magnetic resonance imaging (MRI) and CT showed no evidence of cerebral haemorrhage (see Supplementary material online, *Fig*ure *1A*) or infarction (see Supplementary material online, *Fig*ure *1B*).

**Table 1:** Laboratory tests

Variables	Value	Refence values
Total protein, g/dL	6.4	6.7-8.3
Albumin, g/dL	2.7	3.9-4.9
AST, IU/L	94	8-38
ALT, IU/L	52	4-44
γ-GTP, IU/L	33	16-73
Total bilirubin, mg/dL	7.3	0.2-1.2
Direct bilirubin, mg/dL	3.0	0-0.4
LDH, IU/L	812	106-211
BUN, mg/dL	60.7	8-20
Cre, mg/dL	1.79	0.4-0.8
Estimated glomerular filtration rate, ml/min/1.73 mm^2^	22	60-100
Na, mEq/L	134	135-147
K, mEq/L	3.8	3.3-4.8
Cl, mEq/L	99	98-108
CK, IU/L	411	45-170
CK-MB, IU/L	40	0-12
Troponin I, pg/mL	19,966	0-26.2
Fasting glucose, mg/dL	254	70-110
Haemoglobin A1c, %	7.0	4.6-6.2
Triglyceride, mg/dL	165	30-150
HDL-C, mg/dL	33.6	41-96
LDL-C, mg/dL	71	0-139
BNP, pg/mL	1306	0-18.4
CRP, mg/dL	35.4	0-1
Haptoglobin		
Type 1-1, mg/dL	<10	43-180
Type 2-1, mg/dL	<10	38-179
Type 2-2, mg/dL	<10	15-119
ADAMTS13 activity, %	34	>10
White blood cell count, /μL	10,800	4500-8000
Seg, %	89.0	-
Stab, %	2.0	-
Eosino, %	6.0	-
Mono, %	1.0	-
Lymph, %	2.0	-
Red blood cell count, *10^4^	333	380-480
Haemoglobin, g/dL	9.4	12-15.2
Haematocrit, %	29.4	34-42
Platelet count, /μL	13,000	12-40
Prothrombin time-international normalized ratio	1.33	
Activated partial thromboplastin time, sec	10.8	25-35
D-dimer, μg/mL	33.9	0-1
Fibrinogen, mg/dL	343	200-400
Hepatitis B virus antigen IU/ml	<0.01	0-0.04
Hepatitis C virus antibody, S/CO	<0.1	0-0.9
Human immunodeficiency virus antibody	(-)	(-)
Human immunodeficiency virus antigen	(-)	(-)

ALT, alanine aminotransferase, AST, aspartate aminotransferase, BNP, brain natriuretic peptide, BUN, blood urea nitrogen, CK, creatin-kinase, CRP, C-reactive protein, GTP, glutamyl transpeptidase, HDL, high-density lipoprotein, LDH, lactate dehydrogenase, LDL, low-density lipoprotein

**Figure 2:**
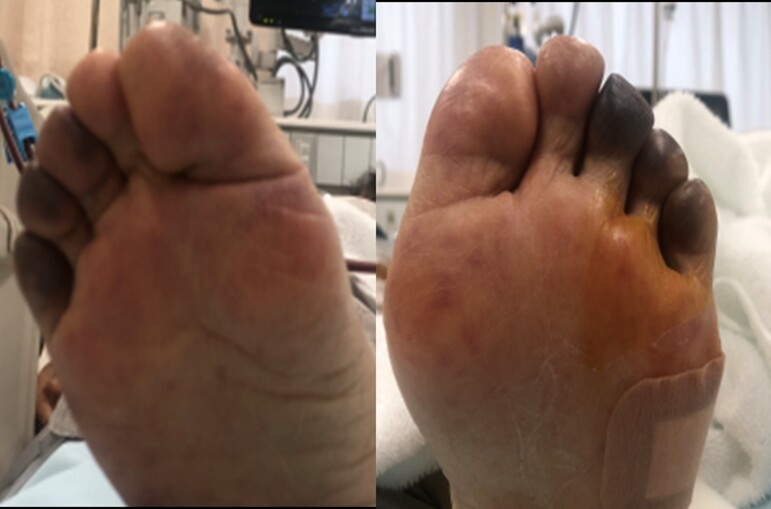
Purpura on both toes. Day 2: purpura on both toes.

Considering the severe thrombocytopaenia, the management of non-ST-elevation myocardial infarction and heart failure was conservative, with non-invasive respiratory support of the BiPAP, carperitide (0.025 μg/kg/mL), and diuretics (furosemide, 20 mg/day), in the intensive care unit. On Day 2, the patient had transient neurologic deficits, including 2/5 weakness in both arms, 2/5 weakness in both legs, dysarthria, and unconsciousness. Given the presence of the classic pentad of TTP, including fever, thrombocytopaenia, haemolytic anaemia, renal dysfunction, and neurologic deficit, the initial diagnosis was TTP, and the patient was managed by a multidisciplinary team including specialists from Cardiology, Haematology, Orthopaedics, and Neurology departments. After administration of FFP infusion, her platelet count decreased to 6000/μL with worsening leg purpura (*Figure 2*). From Day 3 onwards, plasma exchange was performed three times a week. Steroid pulse therapy (1 g/day for 3 days) was administered, followed by high-dose oral steroid therapy (prednisolone 40 mg/day). On Day 5, oral aspirin (100 mg/day) was administered, after recovery of her platelet count to >50 000/µL.^5^

**Figure 3:**
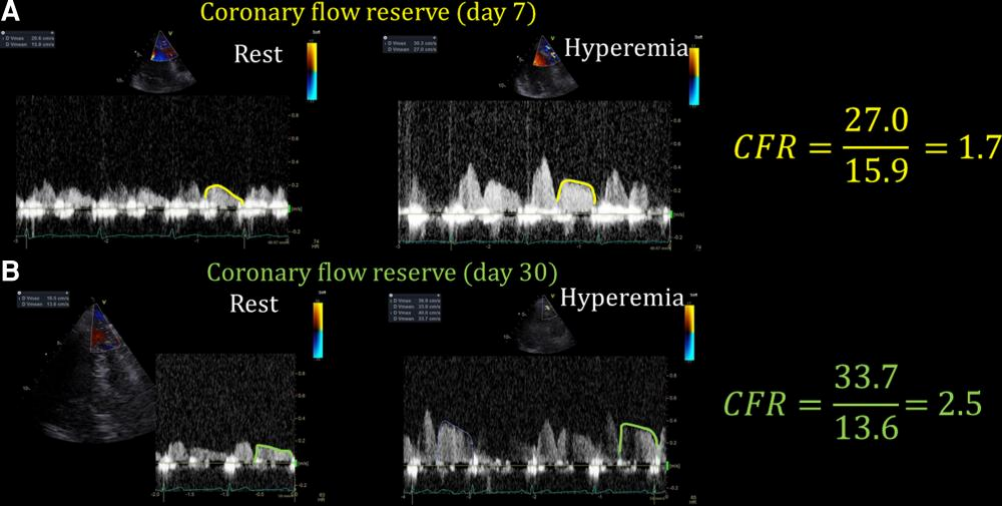
Coronary flow reserve measured using transthoracic Doppler echocardiography at baseline and follow-up. The CFR value was calculated as the ratio of the baseline mean diastolic flow velocity at baseline to the mean diastolic flow velocity at hyperaemia.^6^ (*A*) Impaired coronary flow reserve in the left anterior descending artery at baseline, suggesting coronary microvascular dysfunction caused by thrombotic microangiopathy. (*B*) Serial assessment of coronary flow reserve on Day 30 demonstrated an improvement. There was no significant ST-T change on electrocardiogram during follow-up, whereas left ventricular wall motion was improved after intensive care, including management of heart failure.

**Figure 4:**
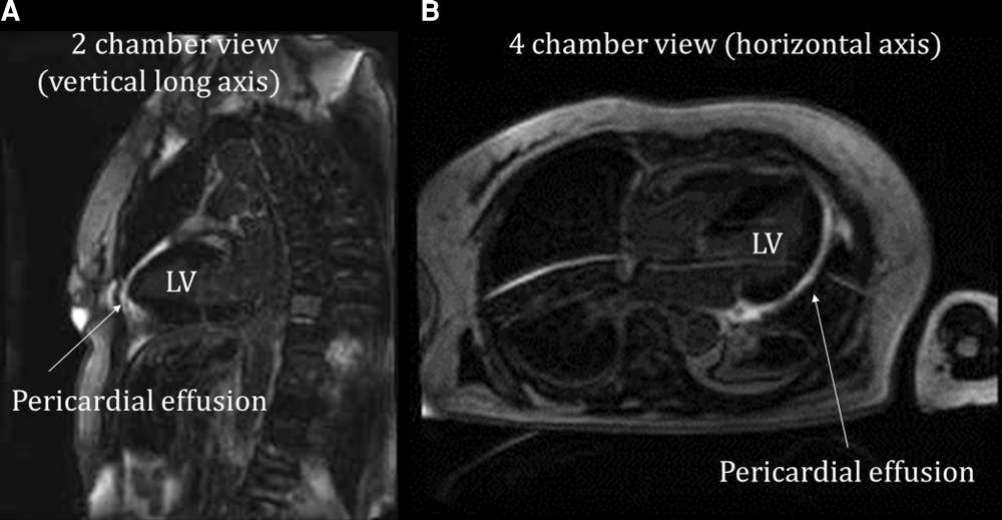
Cardiac magnetic resonance images. (*A*, *B*) Late gadolinium enhancement with cardiac magnetic resonance imaging showed no acute infarct area in the myocardium of the left ventricle. Abnormal left ventricular kinesis as well as ST-T change on electrocardiogram was improved at the time of cardiac magnetic resonance. The cardiac magnetic resonance images revealed pericardial effusion.

Spine MRI was performed to investigate the underlying reason for the back pain and weakness of extremities. The MRI revealed an iliopsoas abscess due to methicillin-resistant coagulase-negative staphylococci, supporting our diagnosis of TTP/TMA caused by infection. In addition, the patient had discitis, potentially caused by the iliopsoas abscess. We evaluated her microcirculation using TTDE, which showed impaired CFR in the left anterior descending artery (LAD; *Figure 3A*), suggesting CMD caused by TTP/TMA. On Day 10, a cardiac MRI showed no evidence of an acute infarct area in the left ventricular myocardium (*Figure 4A*and*B*). Plasma exchange was stopped based on the recovery of platelet count (21.3 × 104/count; LDH 263 IU/L) on Day 21, and corticosteroid therapy was tapered. The patient was haemodynamically stable without symptoms of unconsciousness and recovered from TTP/TMA. On Day 22, coronary angiography was performed to exclude significant obstructive CAD. Coronary angiography revealed no significant luminal stenosis in the LAD and left circumflex artery (see Supplementary material online, *Fig*ure *2A*), while the right coronary artery was small (the left dominance of coronary arteries is shown in Supplementary material online, *Figure 2B*). A follow-up assessment of the CFR on Day 30 (*Figure 3B*) demonstrated an improvement (CFR = 2.5), indicating recovery of the transient impaired CMD as well as improvement in thrombocytopaenia. ADAMTS13 activity (Day 4) was preserved (34%, 2 weeks after the test), and the diagnosis was TMA. Oral prednisolone was gradually reduced, and the iliopsoas abscess was treated with antibiotics for 3 months. The patient was discharged without any symptom of heart or renal failure, and continued the rehabilitation for several months because of a drop in her activities of daily living.

## Discussion

Coronary microvascular dysfunction is the mechanism underlying myocardial infarction/injury with or without epicardial obstructive coronary artery stenosis.^4^ TMA is a spectrum of life-threatening syndromes, in which haemolytic anaemia and thrombocytopaenia are associated with multiple organ damage.^1,2^ Complications include acute myocardial infarction/injury as the major cause of death in patients with TMA. Cardiovascular system complications can be explained by microvascular occlusions due to platelet aggregation. To the best of our knowledge, this is the first report of CMD evaluation and its recovery in TMA.

ADAMTS13 deficiency causes TTP and is diagnosed when ADAMTS13 activity is markedly reduced (<10%).^1^ This contributes to the formation of microthrombi and von Willebrand factor multimers mediating platelet plugs. HUS caused by Shiga toxin-producing *Escherichia coli* (STEC) is the most common cause of TMA associated with infectious diseases (STEC-HUS).^1^ Complement-mediated TMA is atypical HUS and includes coagulation-related TMA due to abnormalities in the thrombomodulin gene. Several underlying conditions (autoimmune diseases, pregnancy, and haematopoietic stem-cell transplantation) can also contribute to secondary TMA.^1^ Disseminated intravascular coagulation should be considered in the differential diagnosis of TMA; however, it is difficult to differentiate given the overlap at the end stage of the disease. Samuel *et al*.^7^ showed that the initial normal ADAMTS13 activity may fall substantially by 2 weeks. In our case, ADAMTS13 activity was preserved despite the typical TTP symptoms. This may be partially explained by the influence of FFP infusion on the measurements for ADAMTS13 activity.

Thrombotic microangiopathy is a pathological condition caused by endothelial cell damage in the small terminal arteries and capillaries, as a result of complete or partial occlusion caused by platelet and hyaline thrombi. This induces CMD and myocardial ischaemia, leading to myocardial infarction (23.4%), heart failure (15.3%), and sudden cardiac death (7.2%).^8^ Approximately 60% of patients with TTP have elevated cardiac troponin level; however, many patients are asymptomatic. Cardiac troponin I level >0.25 μg/L is an independent factor of a three-fold increased risk of death or refractoriness in these patients, indicating that this level of cardiac troponin I can be a prognostic factor.^9^

Persistent CMD can be observed via pathogenic and physiologic mechanisms.^4,6^ CMD recovery is necessary for understanding the natural history of cardiac involvement caused by myocardial infarction/injury in TMA. Moreover, persistent elevated cardiac troponin level could be a useful marker for predicting short- and long-term mortality in various clinical settings, including in patients with renal dysfunction.^10^ TMA with severe renal failure is often treated with plasma exchange, which artificially reduces troponin level. CFR assessed by TTDE was found to be associated with poorer CV outcomes in patients with chronic kidney disease,^11^ serving as an alternative marker for predicting TMA outcomes. Although plasma exchange is the first-line therapy recommended for patients with TTP, plasma exchange was not started immediately after TTP diagnosis in our case.^5^ Some cases do not meet the diagnostic criteria for TTP despite the presence of typical features, yet effectively respond to plasma exchange therapy. Similar to the present case, these cases are considered analogous to TTP,^1^ and conventional plasma exchange and supportive care using FFP could be useful. In addition to standard therapy including plasma exchange and steroid therapy, pharmacological agents such as caplacizumab and rituximab have been reported to be effective in patients with TTP accompanied by cardiac involvement.^5^

Our case demonstrates the importance of identifying the recovery of the coronary microvascular function, as it could be a predictive factor in TMA. One possible explanation for the negative CMR findings in our case is the relatively low diagnostic accuracy of the late gadolinium enhancement in detecting myocardial infarction/damage in patients with MINOCA.^12^ The European Society of Cardiology guideline indicates that TTDE of the LAD, CMR, and PET may be considered for the non-invasive assessment of CFR (Class IIb) in patients with suspected coronary microvascular angina.^3^ Although CFR assessment with TTDE is a cost-effective, non-invasive method utilized in daily clinical practice, the serial measurements should be interpreted with caution given their limited reproducibility.^13^ Non-invasive assessment of CMD with MRI or positron emission tomography/CT was not available at our institution; however, it could be a useful diagnostic tool for evaluating CMD.^4^

## Conclusion

*In vivo* evaluations of coronary microvascular function can provide mechanistic insights into the cardiac involvement in TMA. Further studies are warranted to determine whether non-invasive assessment of microvascular function enables disease progression predictions and provides prognostic implications beyond cardiac troponin elevation in patients with TMA.

## Supplementary Material

ytac318_Supplementary_Data

## Lead author biography

Kenichiro Otsuka was born in 1980 in Kyoto, Japan. He received his medical training at the Osaka City University Hospital. In 2014, he graduated from Osaka City University Graduate School of Medicine. He trained as a research fellow in Massachusetts general Hospital, Harvard Medical School in Boston, USA. His research focus has been investigation of *in vivo* vascular biology using OCT-polarimetry to better understand the mechanisms of acute coronary syndromes.

## Supplementary material

**Slide sets:** A fully edited slide set detailing this case and suitable for local presentation is available online as Supplementary data.

**Consent:** The authors confirm that written consent for the submission and publication of this case report, including images and associated text, has been obtained from the patient, in line with COPE guidance.

**Funding:** None declared.

### Data availability

The datasets used and analyzed during the current study are available from the corresponding author on reasonable request.
