# Supplementary material for: Evaluations of coronary microvascular dysfunction in a patient with thrombotic microangiopathy and cardiac troponin elevation: a case report
Source: Eur Heart J Case Rep. 2022 Jul 29;7(3):ytac318. doi: 10.1093/ehjcr/ytac318 (PMC10020975; doi:10.1093/ehjcr/ytac318)

Supplementary materials

Supplemental figure 1. Brain imaging on admission.

Figure 1a. Brain computed tomography showed no evidence of cerebral infarction or haemorrhage.

Figure 1b. Brain magnetic resonance imaging showed no evidence of cerebral infarction or haemorrhage.

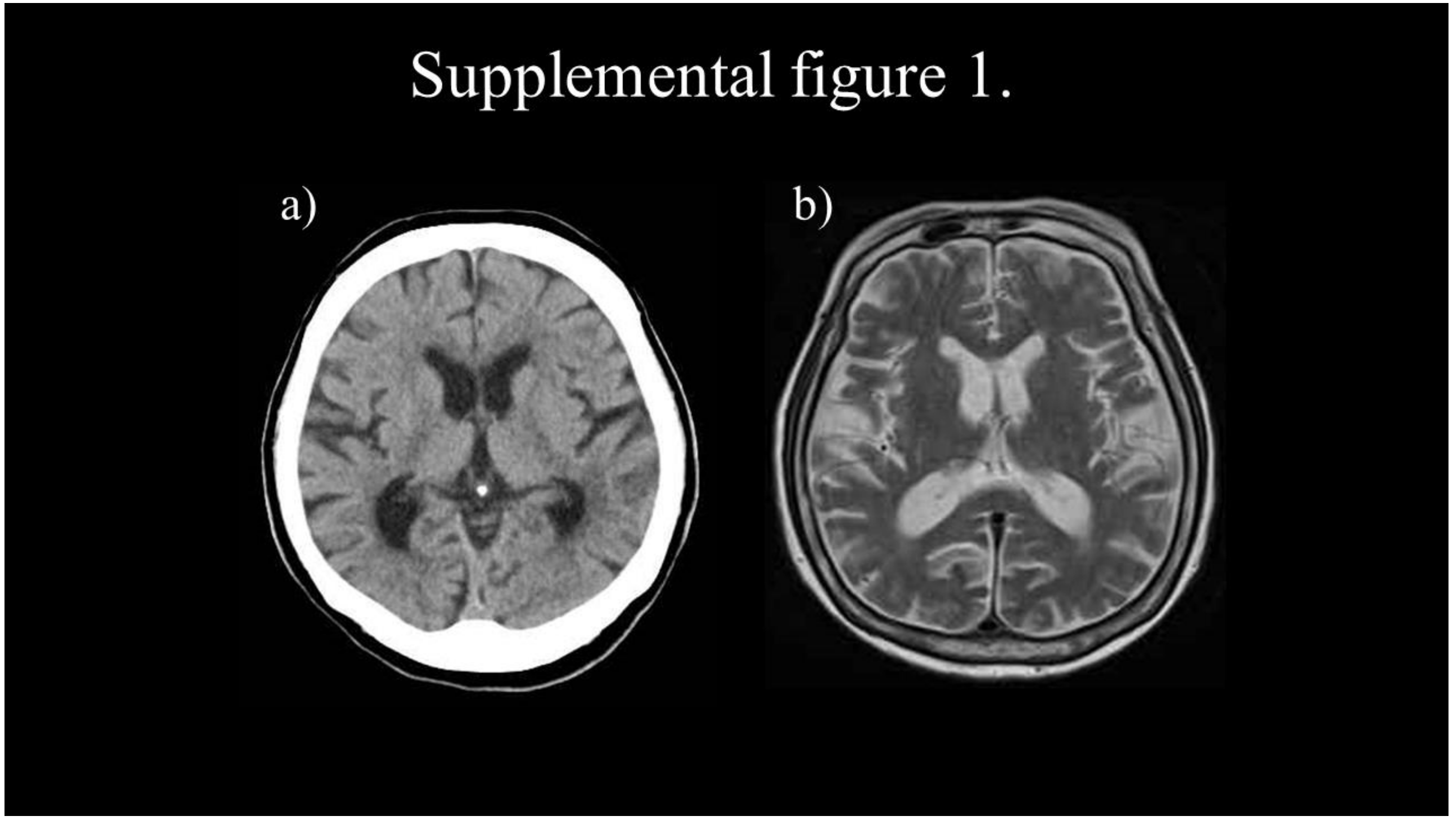

Supplementary figure 2. Invasive coronary angiography: Coronary angiography (day 22) revealed no significant luminal stenosis in the left anterior descending artery and left circumflex artery (a), while the right coronary artery was small (b).

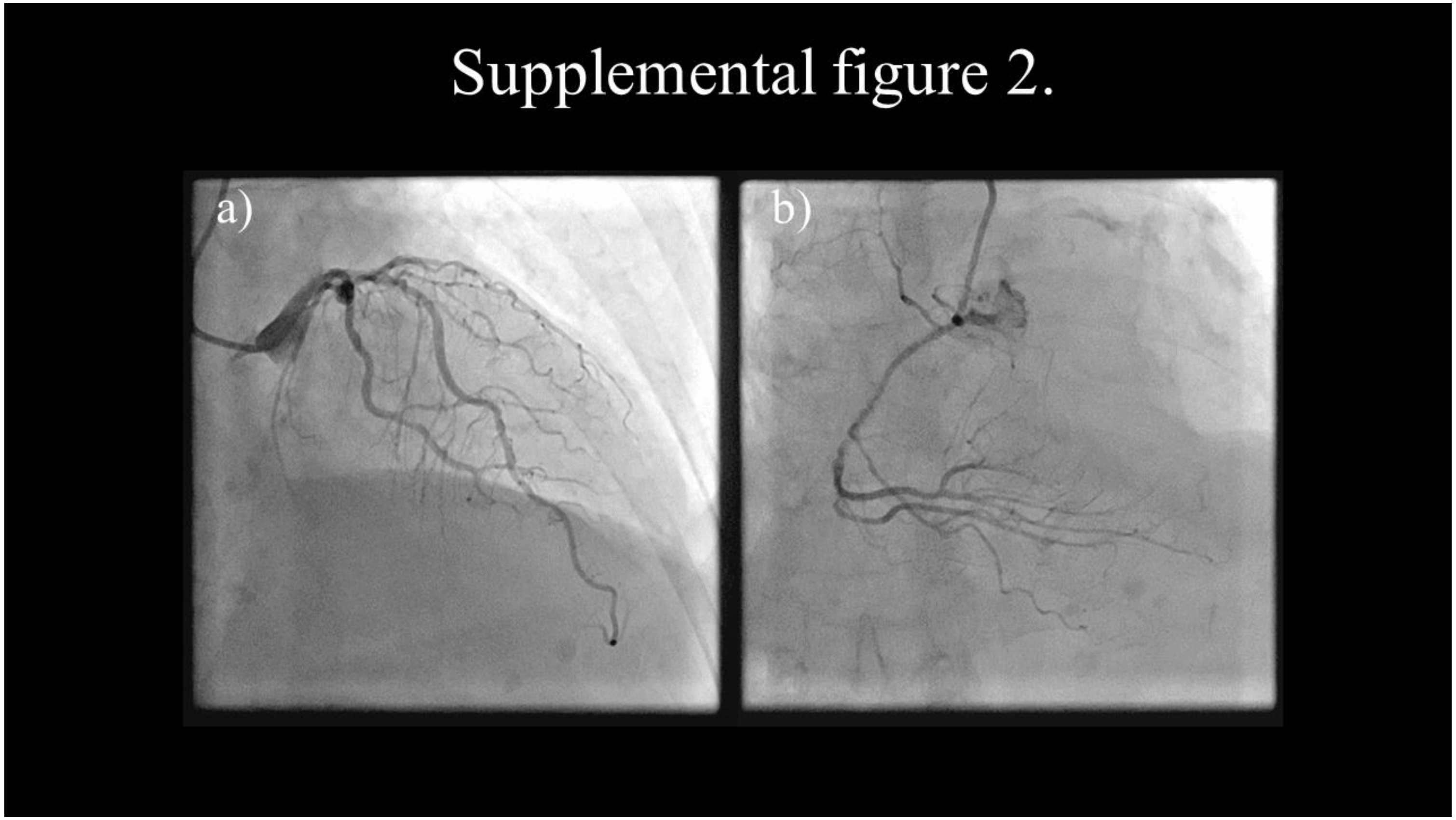

Supplementary figure 3. This figure illustrates changes of laboratory tests, including hemoglobin, platelet count, total bilirubin, CRP, eGFR, CK-MB, and cardiac troponin I.

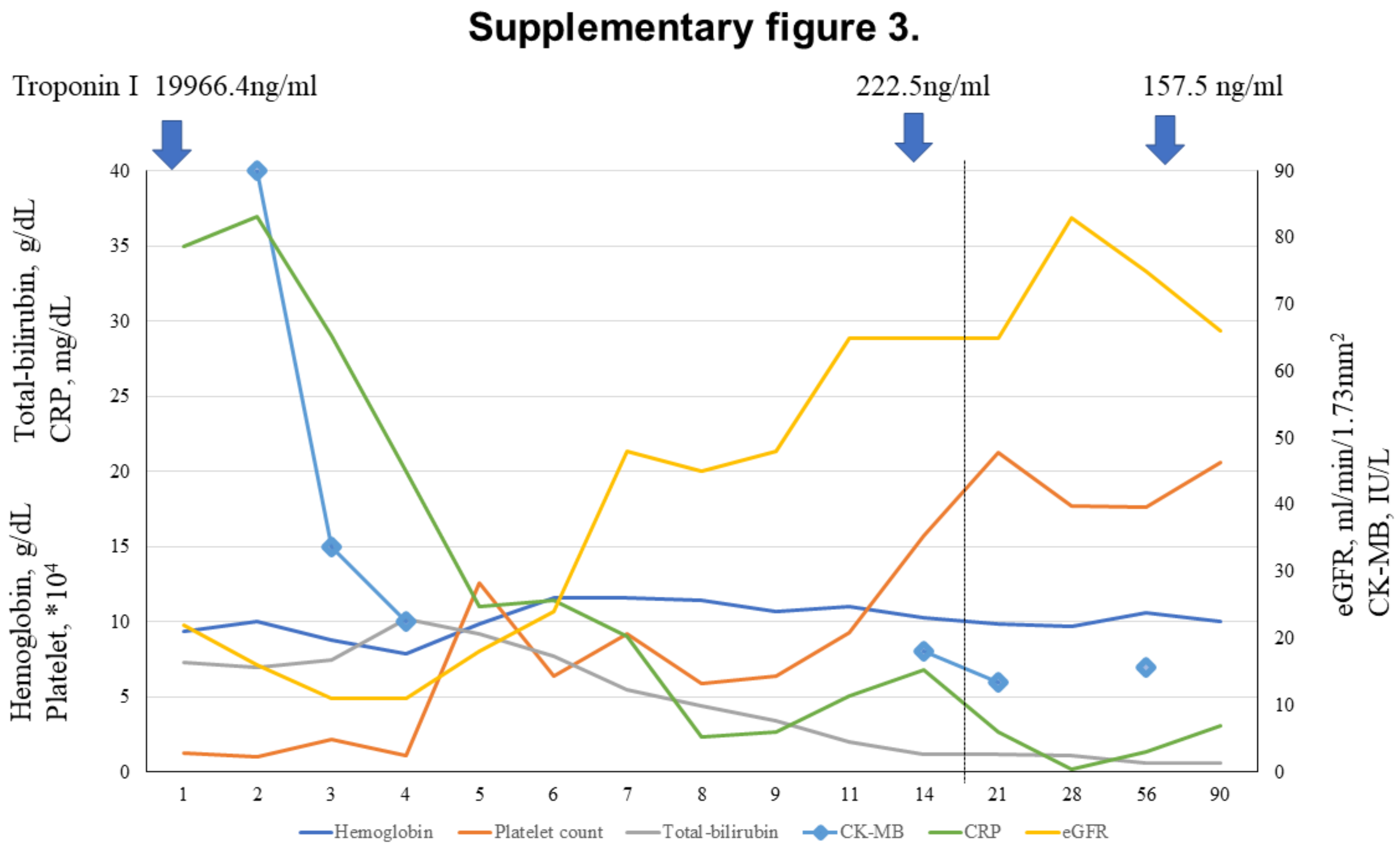

Supplement: ytac318_Supplementary_Data [file ytac318_supplementary_data.zip › Supplementary_materials.pdf]
