# Supplementary material for: Evaluations of coronary microvascular dysfunction in a patient with thrombotic microangiopathy and cardiac troponin elevation: a case report
Source: Eur Heart J Case Rep. 2022 Jul 29;7(3):ytac318. doi: 10.1093/ehjcr/ytac318 (PMC10020975; doi:10.1093/ehjcr/ytac318)
Supplement: ytac318_Supplementary_Data [file ytac318_supplementary_data.zip › Slideset.pptx]

## Slide 1
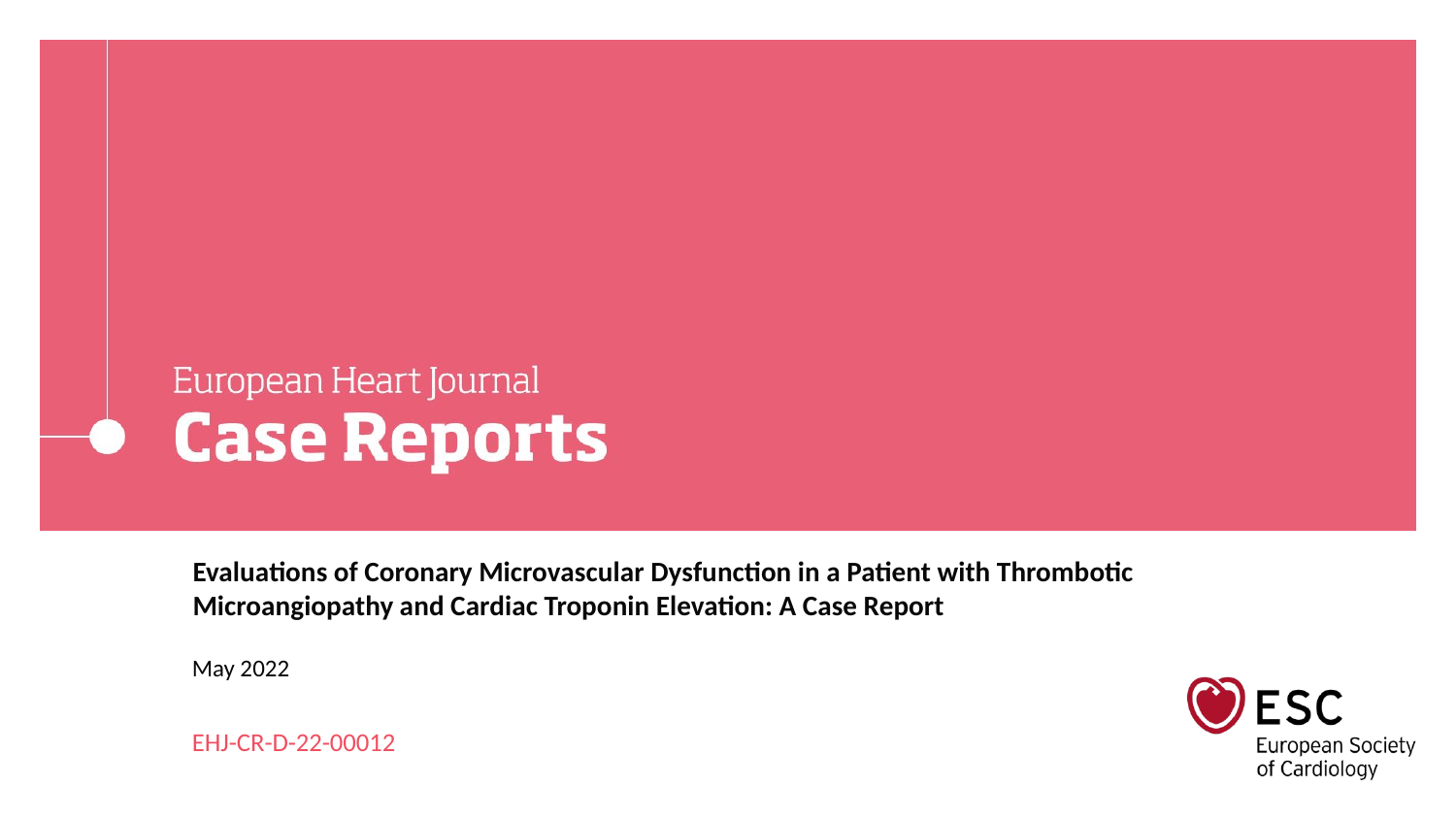

# Evaluations of Coronary Microvascular Dysfunction in a Patient with Thrombotic Microangiopathy and Cardiac Troponin Elevation: A Case Report
May 2022
EHJ-CR-D-22-00012

## Slide 2
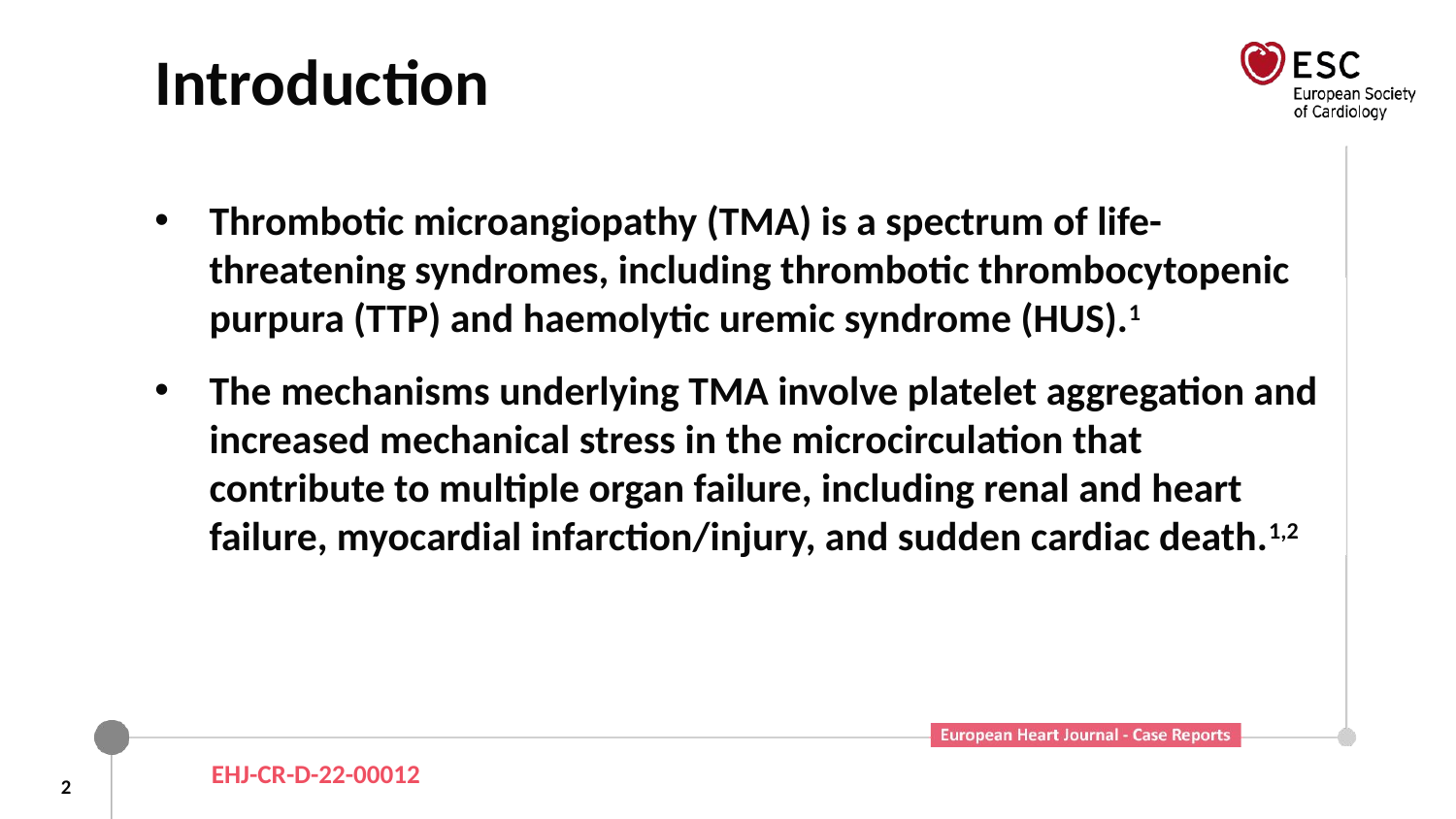

# Introduction
Thrombotic microangiopathy (TMA) is a spectrum of life-threatening syndromes, including thrombotic thrombocytopenic purpura (TTP) and haemolytic uremic syndrome (HUS).1
The mechanisms underlying TMA involve platelet aggregation and increased mechanical stress in the microcirculation that contribute to multiple organ failure, including renal and heart failure, myocardial infarction/injury, and sudden cardiac death.1,2
EHJ-CR-D-22-00012
2

## Slide 3
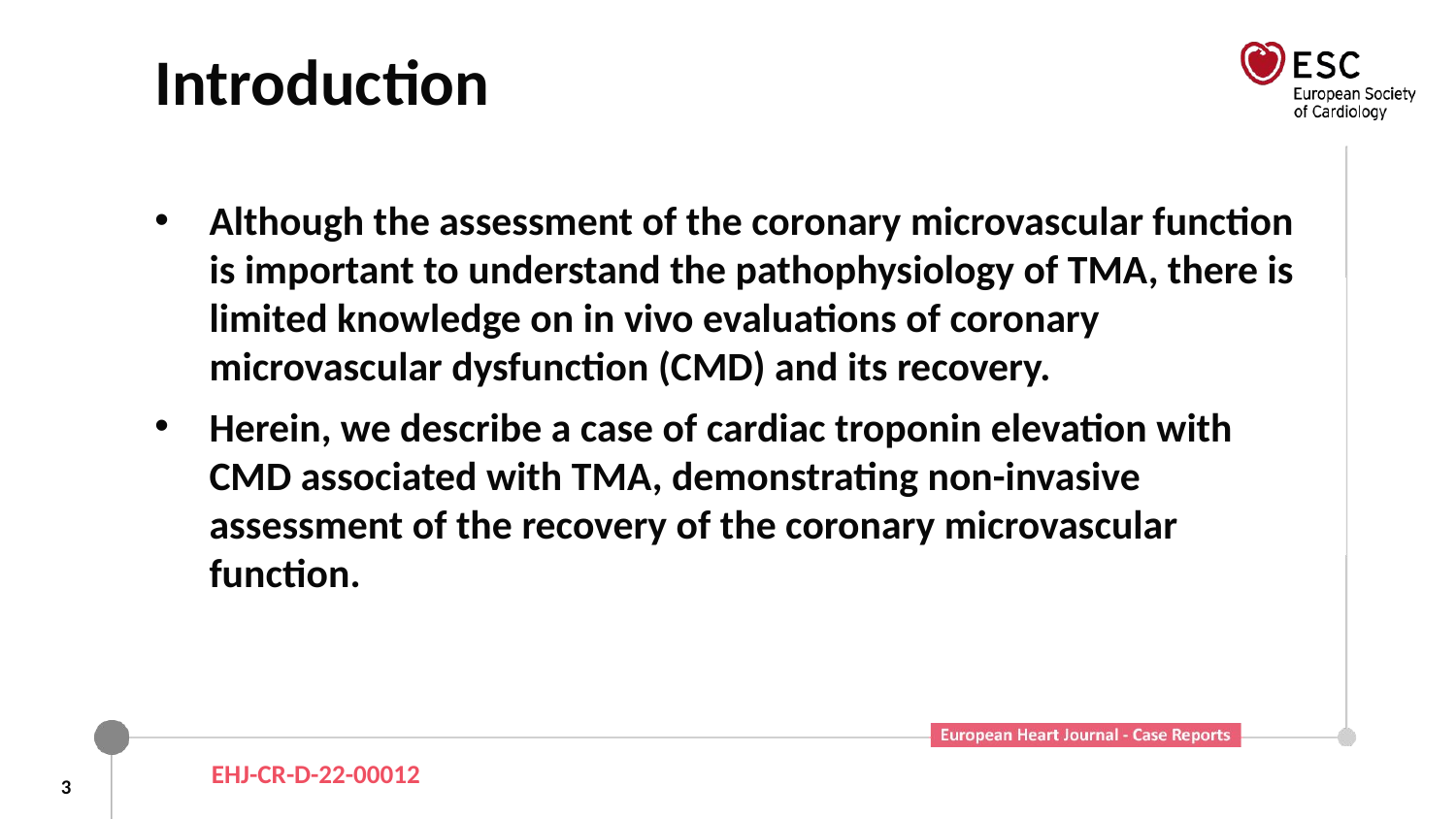

# Introduction
Although the assessment of the coronary microvascular function is important to understand the pathophysiology of TMA, there is limited knowledge on in vivo evaluations of coronary microvascular dysfunction (CMD) and its recovery.
Herein, we describe a case of cardiac troponin elevation with CMD associated with TMA, demonstrating non-invasive assessment of the recovery of the coronary microvascular function.
EHJ-CR-D-22-00012
3

## Slide 4
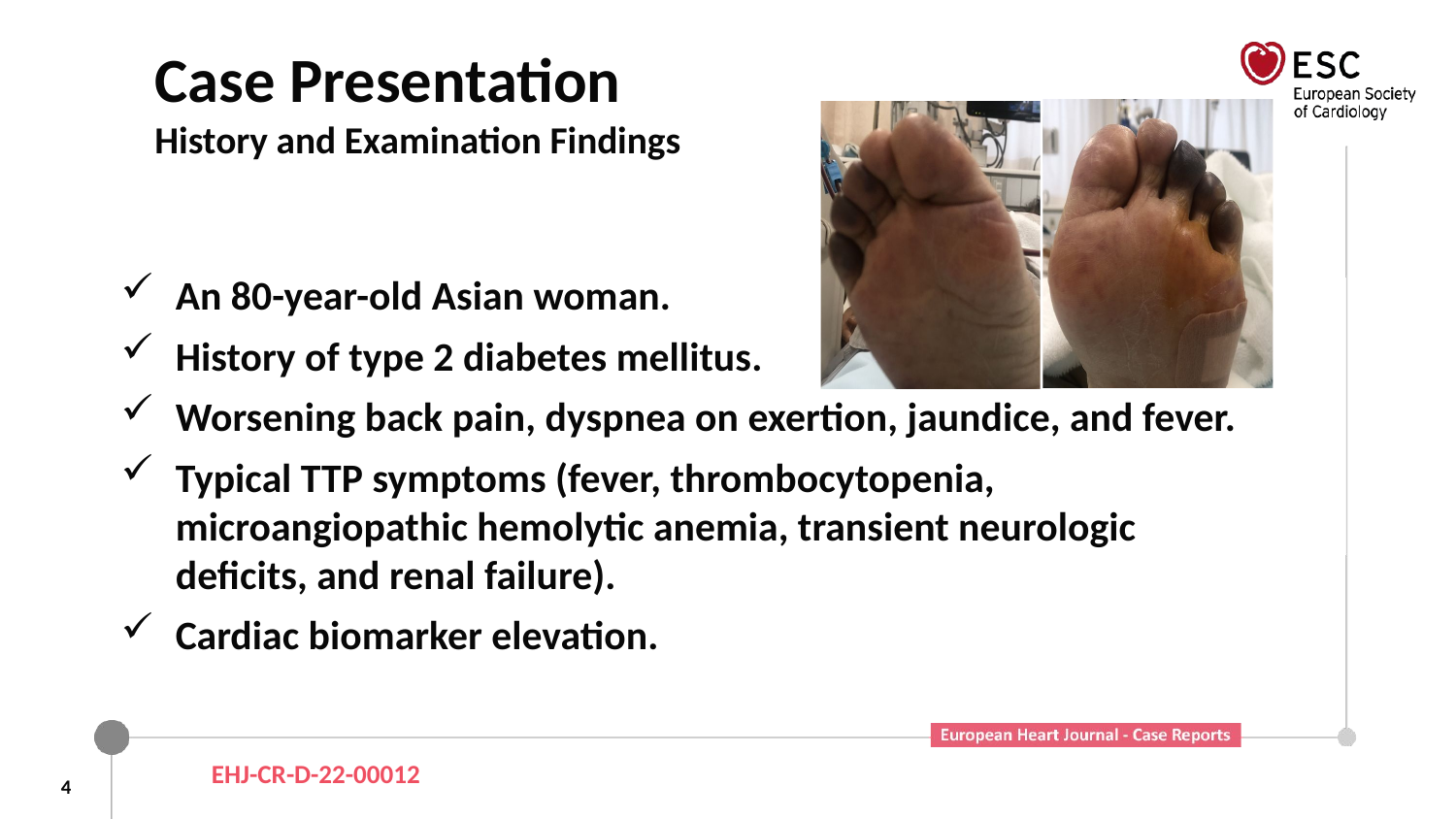

# Case Presentation History and Examination Findings
An 80-year-old Asian woman.
History of type 2 diabetes mellitus.
Worsening back pain, dyspnea on exertion, jaundice, and fever.
Typical TTP symptoms (fever, thrombocytopenia, microangiopathic hemolytic anemia, transient neurologic deficits, and renal failure).
Cardiac biomarker elevation.
EHJ-CR-D-22-00012
4

## Slide 5
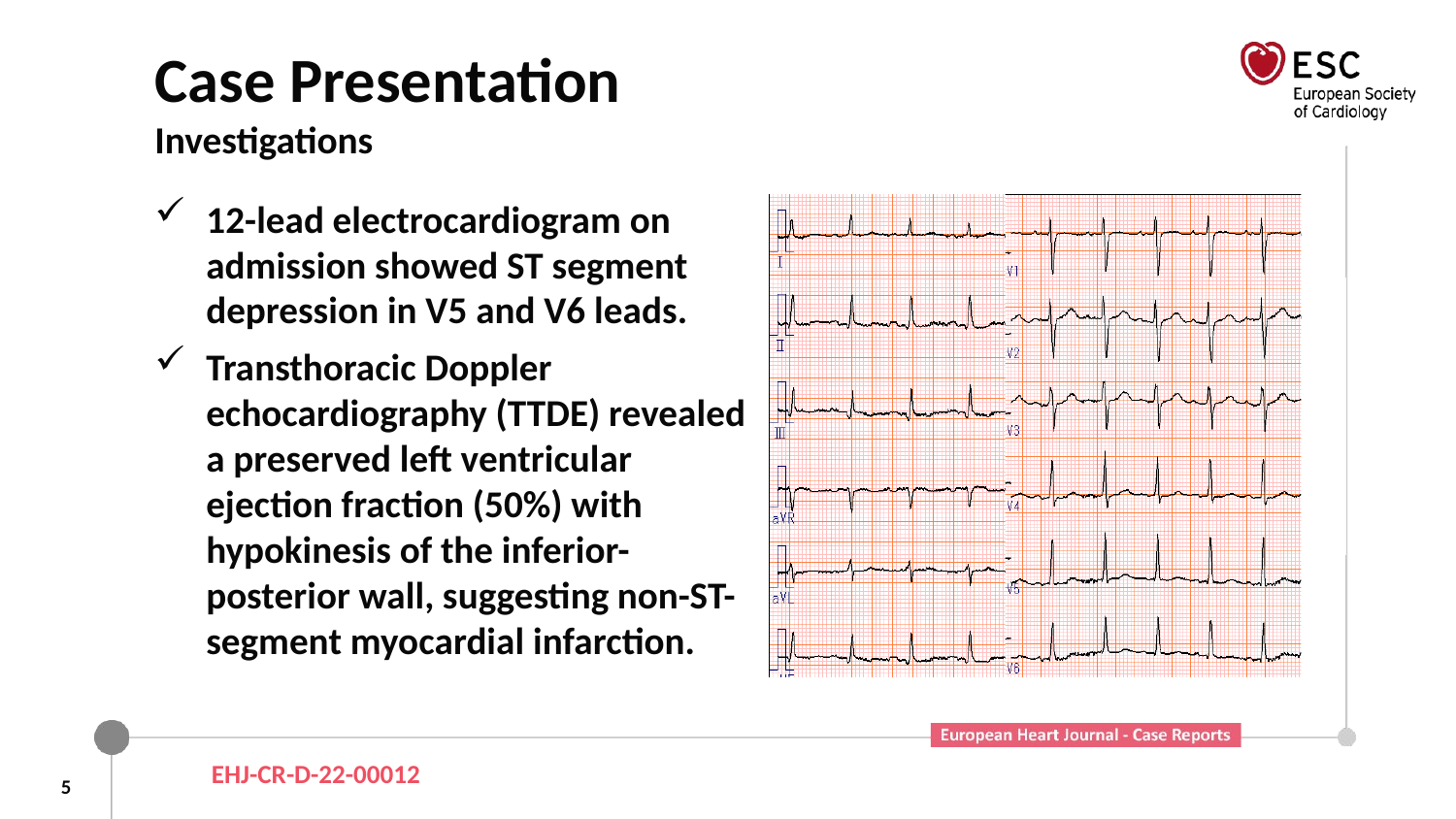

# Case Presentation Investigations
12-lead electrocardiogram on admission showed ST segment depression in V5 and V6 leads.
Transthoracic Doppler echocardiography (TTDE) revealed a preserved left ventricular ejection fraction (50%) with hypokinesis of the inferior-posterior wall, suggesting non-ST-segment myocardial infarction.
EHJ-CR-D-22-00012
5

## Slide 6
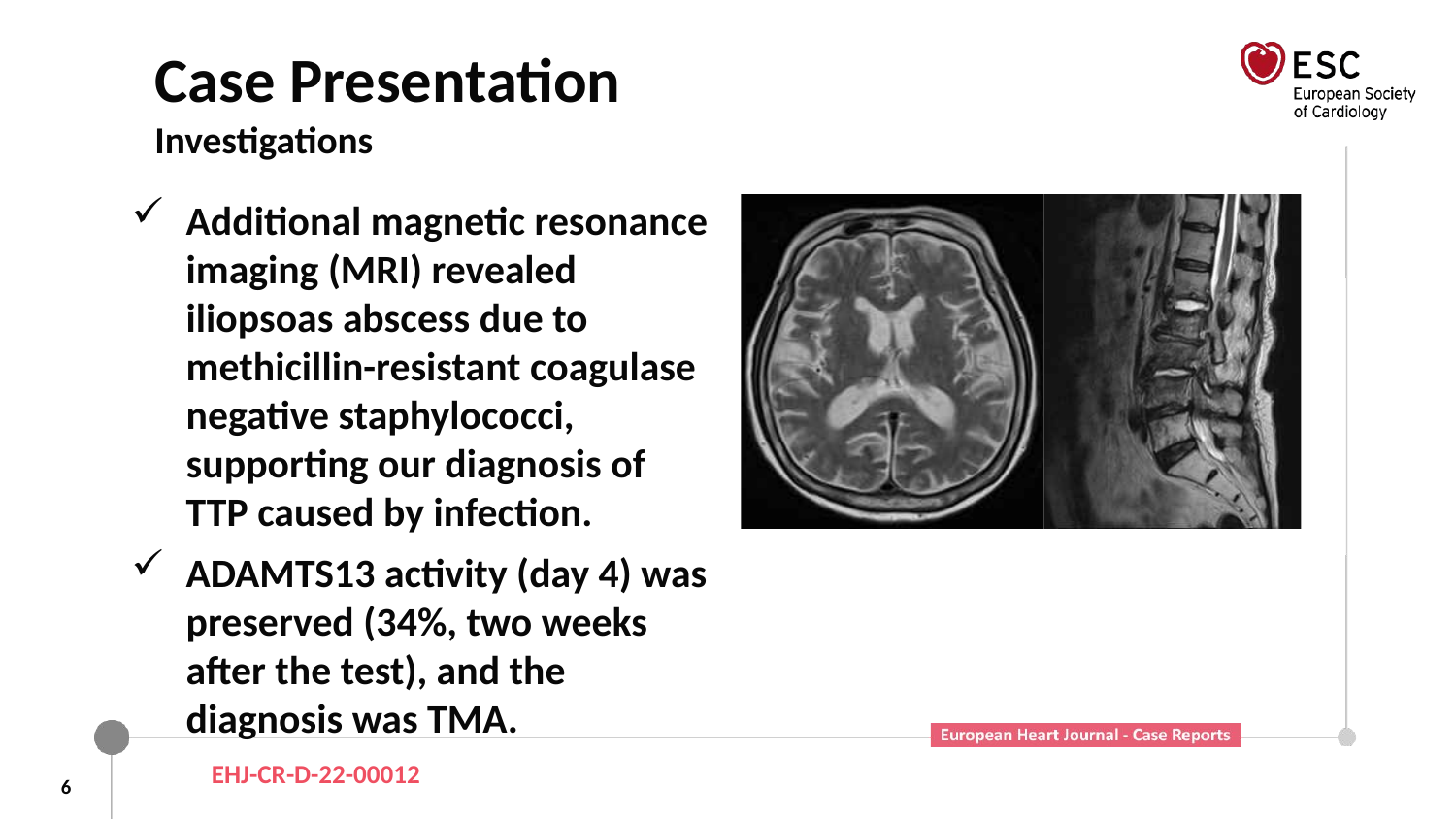

# Case Presentation Investigations
Additional magnetic resonance imaging (MRI) revealed iliopsoas abscess due to methicillin-resistant coagulase negative staphylococci, supporting our diagnosis of TTP caused by infection.
ADAMTS13 activity (day 4) was preserved (34%, two weeks after the test), and the diagnosis was TMA.
EHJ-CR-D-22-00012
6

## Slide 7
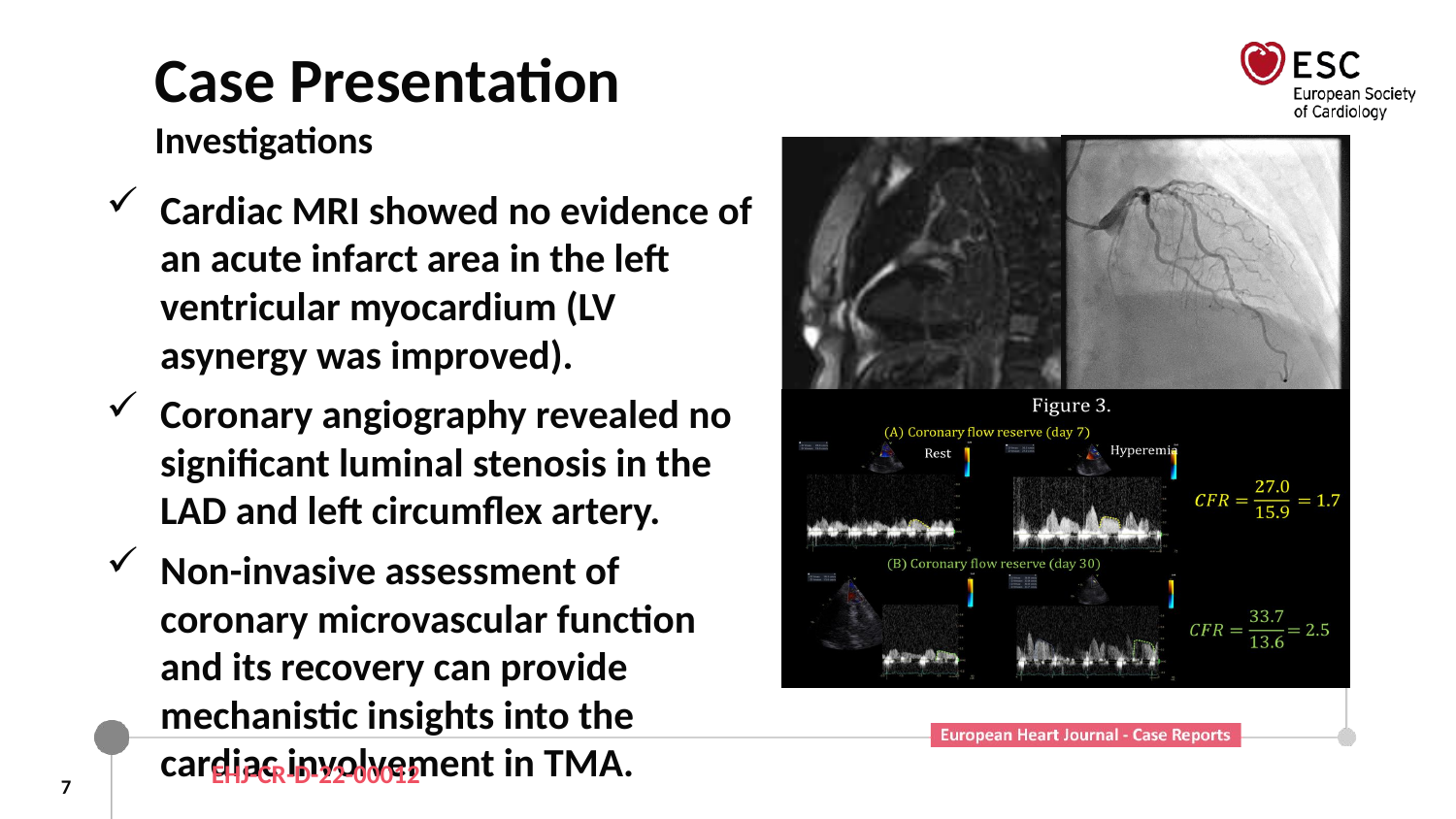

# Case Presentation Investigations
Cardiac MRI showed no evidence of an acute infarct area in the left ventricular myocardium (LV asynergy was improved).
Coronary angiography revealed no significant luminal stenosis in the LAD and left circumflex artery.
Non-invasive assessment of coronary microvascular function and its recovery can provide mechanistic insights into the cardiac involvement in TMA.
EHJ-CR-D-22-00012
7

## Slide 8
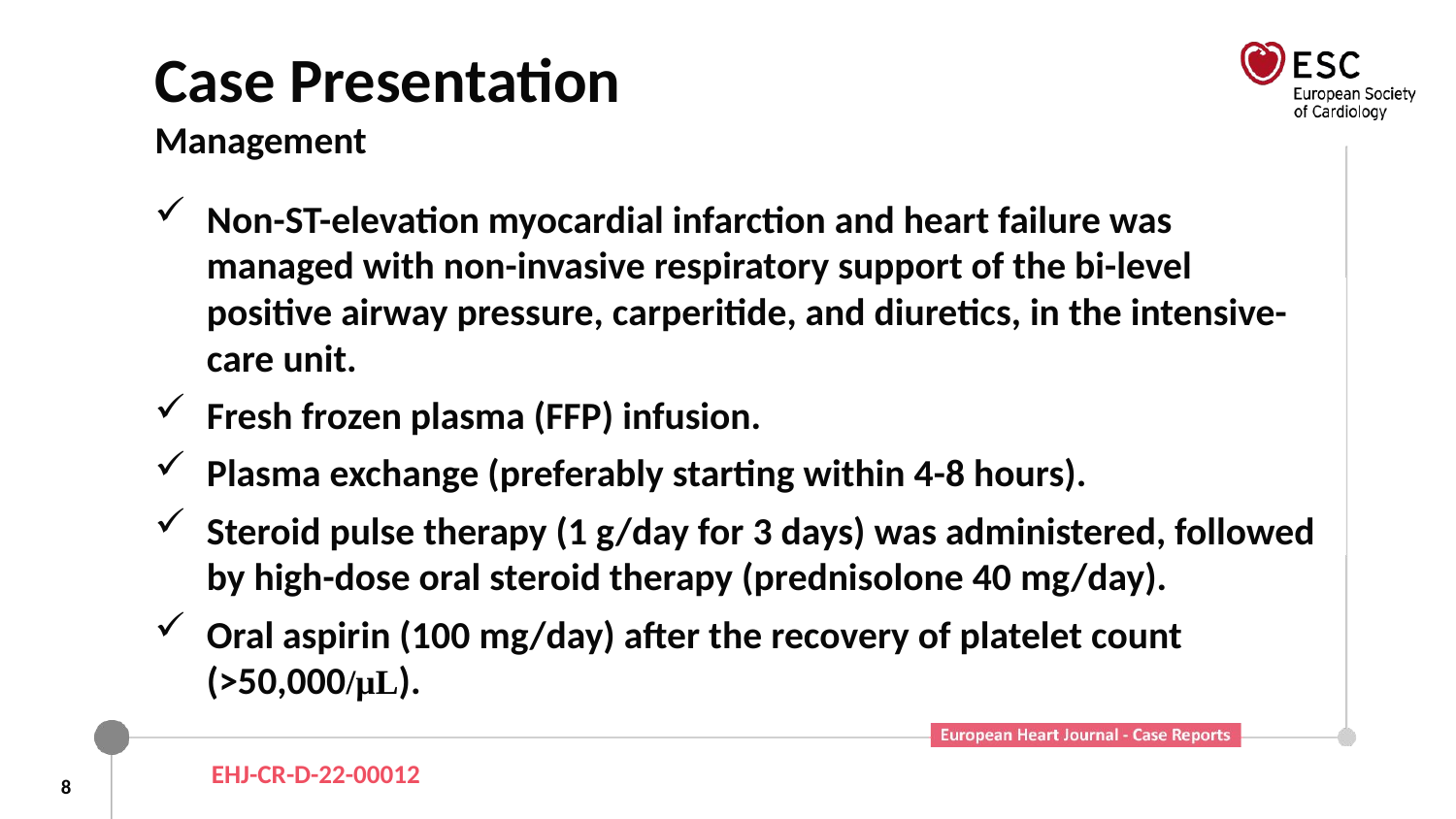

# Case Presentation Management
Non-ST-elevation myocardial infarction and heart failure was managed with non-invasive respiratory support of the bi-level positive airway pressure, carperitide, and diuretics, in the intensive-care unit.
Fresh frozen plasma (FFP) infusion.
Plasma exchange (preferably starting within 4-8 hours).
Steroid pulse therapy (1 g/day for 3 days) was administered, followed by high-dose oral steroid therapy (prednisolone 40 mg/day).
Oral aspirin (100 mg/day) after the recovery of platelet count (>50,000/µL).
EHJ-CR-D-22-00012
8

## Slide 9
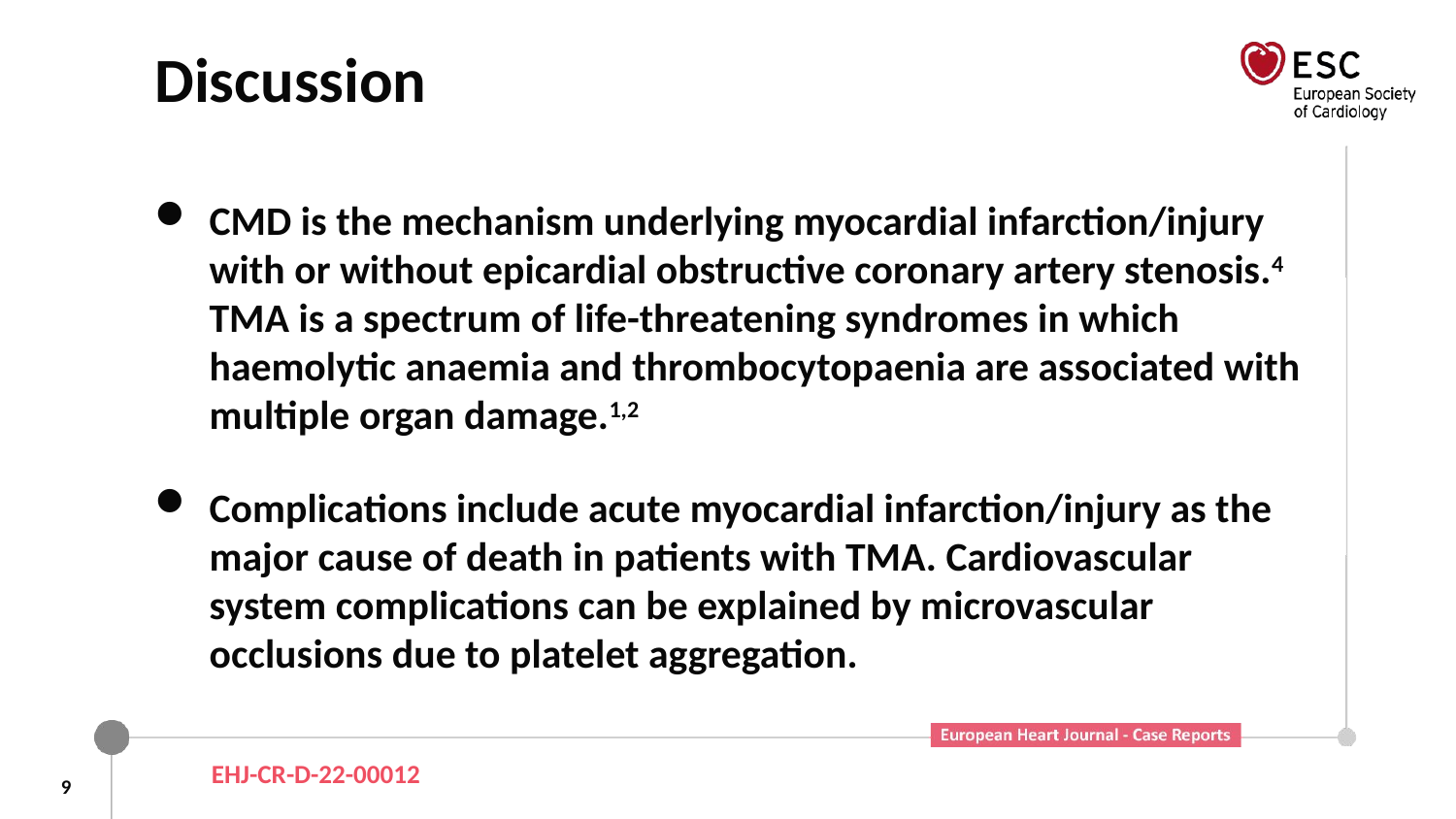

# Discussion
CMD is the mechanism underlying myocardial infarction/injury with or without epicardial obstructive coronary artery stenosis.4 TMA is a spectrum of life-threatening syndromes in which haemolytic anaemia and thrombocytopaenia are associated with multiple organ damage.1,2
Complications include acute myocardial infarction/injury as the major cause of death in patients with TMA. Cardiovascular system complications can be explained by microvascular occlusions due to platelet aggregation.
EHJ-CR-D-22-00012
9

## Slide 10
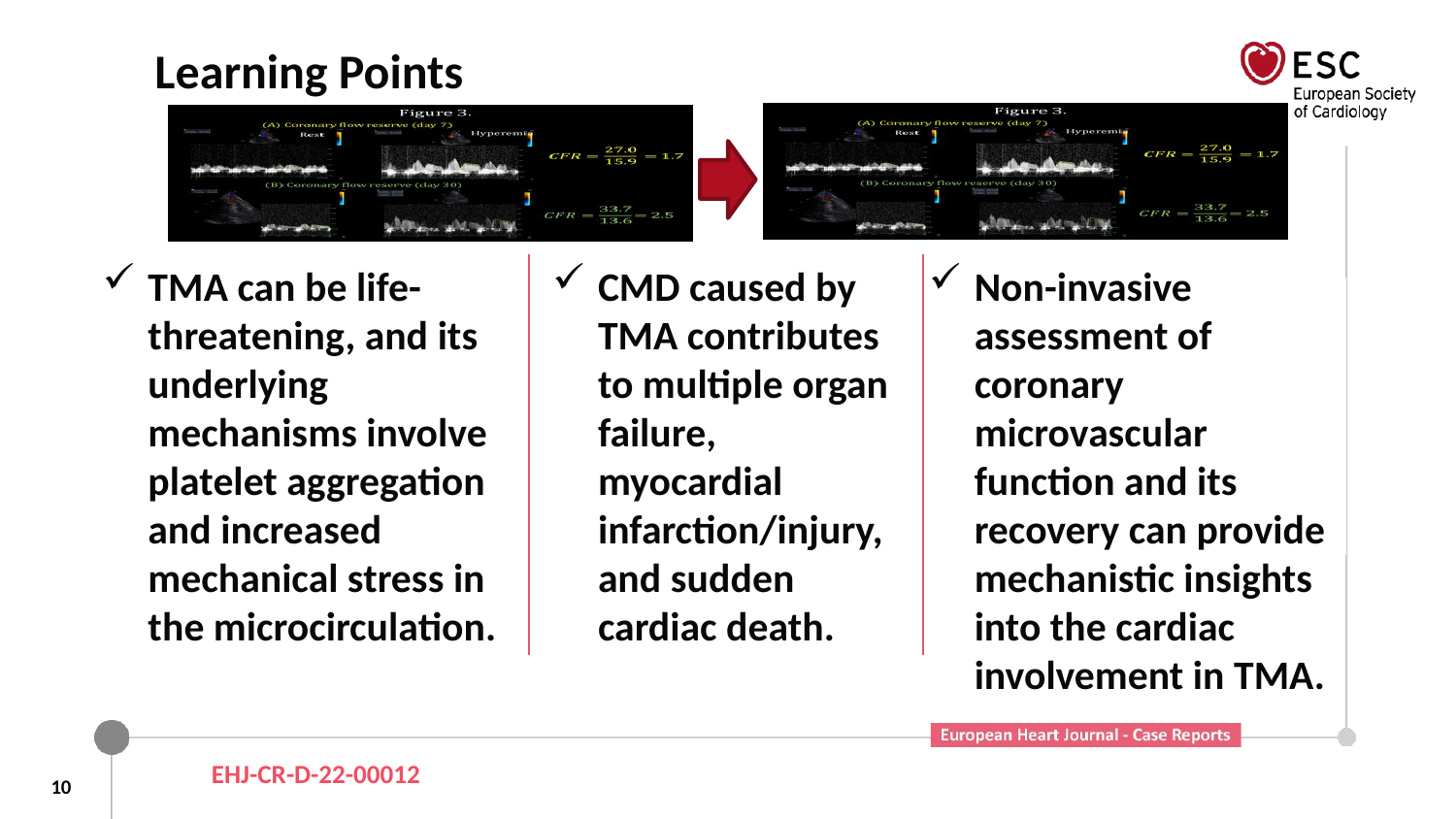

# Learning Points
TMA can be life-threatening, and its underlying mechanisms involve platelet aggregation and increased mechanical stress in the microcirculation.
CMD caused by TMA contributes to multiple organ failure, myocardial infarction/injury, and sudden cardiac death.
Non-invasive assessment of coronary microvascular function and its recovery can provide mechanistic insights into the cardiac involvement in TMA.
EHJ-CR-D-22-00012
10

## Slide 11
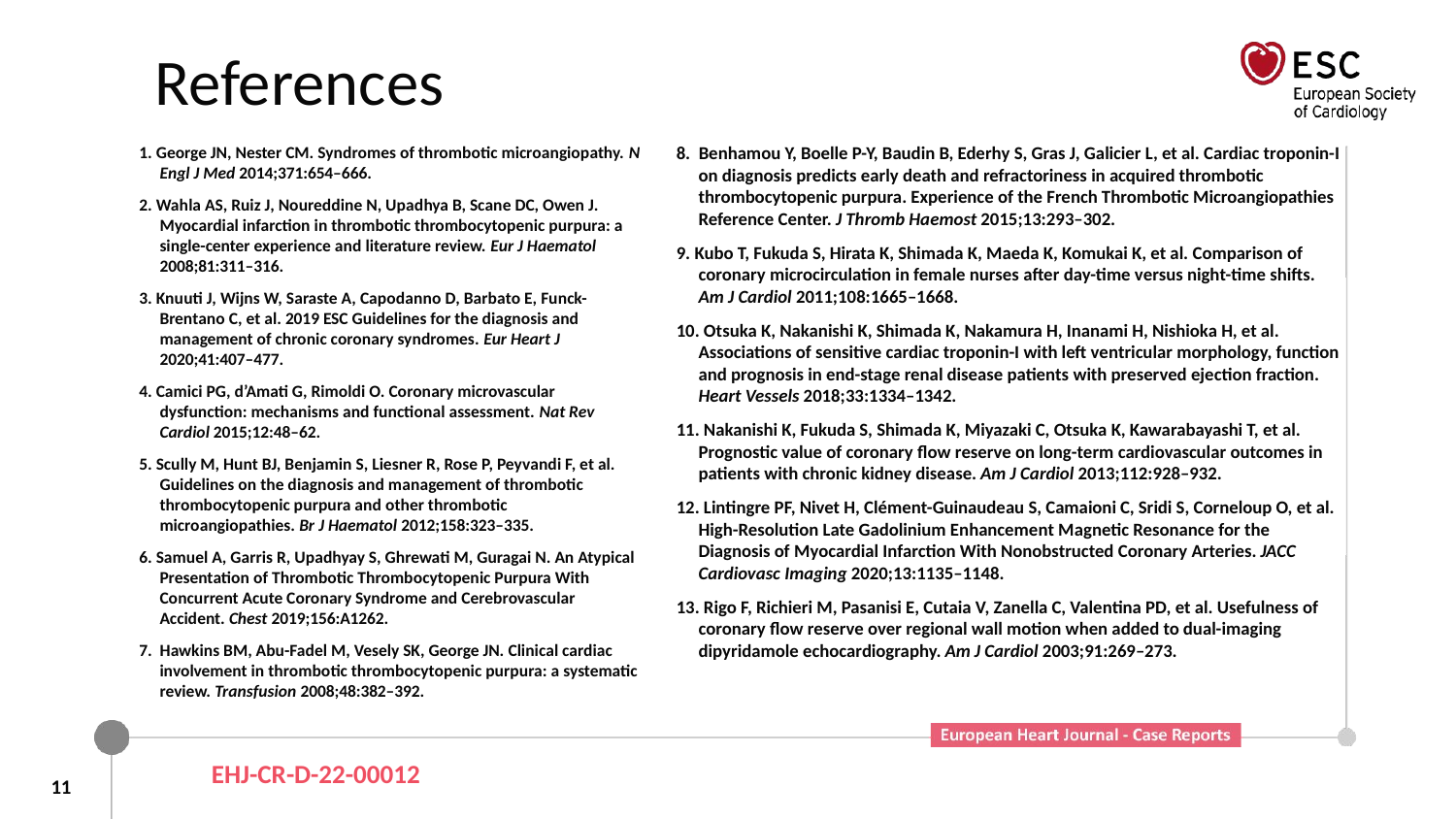

# References
1. George JN, Nester CM. Syndromes of thrombotic microangiopathy. N Engl J Med 2014;371:654–666.
2. Wahla AS, Ruiz J, Noureddine N, Upadhya B, Scane DC, Owen J. Myocardial infarction in thrombotic thrombocytopenic purpura: a single-center experience and literature review. Eur J Haematol 2008;81:311–316.
3. Knuuti J, Wijns W, Saraste A, Capodanno D, Barbato E, Funck-Brentano C, et al. 2019 ESC Guidelines for the diagnosis and management of chronic coronary syndromes. Eur Heart J 2020;41:407–477.
4. Camici PG, d’Amati G, Rimoldi O. Coronary microvascular dysfunction: mechanisms and functional assessment. Nat Rev Cardiol 2015;12:48–62.
5. Scully M, Hunt BJ, Benjamin S, Liesner R, Rose P, Peyvandi F, et al. Guidelines on the diagnosis and management of thrombotic thrombocytopenic purpura and other thrombotic microangiopathies. Br J Haematol 2012;158:323–335.
6. Samuel A, Garris R, Upadhyay S, Ghrewati M, Guragai N. An Atypical Presentation of Thrombotic Thrombocytopenic Purpura With Concurrent Acute Coronary Syndrome and Cerebrovascular Accident. Chest 2019;156:A1262.
7. Hawkins BM, Abu-Fadel M, Vesely SK, George JN. Clinical cardiac involvement in thrombotic thrombocytopenic purpura: a systematic review. Transfusion 2008;48:382–392.
8. Benhamou Y, Boelle P-Y, Baudin B, Ederhy S, Gras J, Galicier L, et al. Cardiac troponin-I on diagnosis predicts early death and refractoriness in acquired thrombotic thrombocytopenic purpura. Experience of the French Thrombotic Microangiopathies Reference Center. J Thromb Haemost 2015;13:293–302.
9. Kubo T, Fukuda S, Hirata K, Shimada K, Maeda K, Komukai K, et al. Comparison of coronary microcirculation in female nurses after day-time versus night-time shifts. Am J Cardiol 2011;108:1665–1668.
10. Otsuka K, Nakanishi K, Shimada K, Nakamura H, Inanami H, Nishioka H, et al. Associations of sensitive cardiac troponin-I with left ventricular morphology, function and prognosis in end-stage renal disease patients with preserved ejection fraction. Heart Vessels 2018;33:1334–1342.
11. Nakanishi K, Fukuda S, Shimada K, Miyazaki C, Otsuka K, Kawarabayashi T, et al. Prognostic value of coronary flow reserve on long-term cardiovascular outcomes in patients with chronic kidney disease. Am J Cardiol 2013;112:928–932.
12. Lintingre PF, Nivet H, Clément-Guinaudeau S, Camaioni C, Sridi S, Corneloup O, et al. High-Resolution Late Gadolinium Enhancement Magnetic Resonance for the Diagnosis of Myocardial Infarction With Nonobstructed Coronary Arteries. JACC Cardiovasc Imaging 2020;13:1135–1148.
13. Rigo F, Richieri M, Pasanisi E, Cutaia V, Zanella C, Valentina PD, et al. Usefulness of coronary flow reserve over regional wall motion when added to dual-imaging dipyridamole echocardiography. Am J Cardiol 2003;91:269–273.
EHJ-CR-D-22-00012
11
